# Oxidative Stress on *Haemonchus contortus* Larvae Exposed to Alternative Treatment with *Artemisia cina n*-Hexane Extract and Cinaguaiacin Metabolites

**DOI:** 10.3390/vetsci12050467

**Published:** 2025-05-14

**Authors:** Ana Elvia Sánchez-Mendoza, Guillermo Reséndiz-González, Eduardo Rico-Mejía, Héctor Alejandro de la Cruz-Cruz, Gerardo Ramírez-Rico, Jorge Alfredo Cuéllar-Ordaz, José Francisco Montiel-Sosa, María Eugenia López-Arellano, David Emmanuel Reyes-Guerrero, Clara Luisa Domínguez-Delgado, Martín Orlando Pulido Medellín, Daniel Hernández-Patlán, Rosa Isabel Higuera-Piedrahita

**Affiliations:** 1 Laboratory 18: Multidisciplinary Research Unit, Superior Studies Faculty at Cuautitlán (FESC), National Autonomous University of Mexico (UNAM), Cuautitlán Izcalli 54714, Mexico; anasanchez12@cuautitlan.unam.mx (A.E.S.-M.); delacruz@unam.mx (H.A.d.l.C.-C.); fmontiel@cuautitlan.unam.mx (J.F.M.-S.); 2 Laboratory 3: Multidisciplinary Research Unit, Superior Studies Faculty at Cuautitlán (FESC), National Autonomous University of Mexico (UNAM), Cuautitlán Izcalli 54714, Mexico; guillermo.resendiz@cuautitlan.unam.mx (G.R.-G.); eduardo.rico.mejia21@comunidad.unam.mx (E.R.-M.); garmvz@gmail.com (G.R.-R.); jcuellar@unam.mx (J.A.C.-O.); 3 Centro Nacional de Investigación Disciplinaria en Salud Animal e Inocuidad, Instituto Nacional de Investigaciones Forestales, Agrícolas y Pecuarias, Jiutepec, Morelos 20440, Mexico; lopez.mariaeugenia@inifap.gob.mx (M.E.L.-A.); reyes.david@inifap.gob.mx (D.E.R.-G.); 4 Laboratory 16: Multidisciplinary Research Unit, Cuautitlán School of Advanced Studies, Superior Studies Faculty at Cuautitlán (FESC), National Autonomous University of Mexico (UNAM), Cuautitlán Izcalli 54714, Mexico; clara_ldd@comunidad.unam.mx; 5 Veterinary Parasitology Laboratory, UPTC, Faculty of Agricultural Sciences, Pedagogical and Technological University of Colombia (UPTC), Tunja-Boyacá 150003, Colombia; martin.pulido@uptc.edu.co; 6 Laboratory 5: LEDEFAR, Multidisciplinary Research Unit, Superior Studies Faculty at Cuautitlan (FESC), National Autonomous University of Mexico (UNAM), Cuautitlan Izcalli 54714, Mexico; 7 Nanotechnology Engineering Division, Polytechnic University of the Valley of Mexico, Tultitlán 54910, Mexico

**Keywords:** *Artemisia cina*, anthelmintics, oxidative stress, alternative treatment, action mechanism

## Abstract

Anthelmintic resistance is currently negatively impacting animal production parameters, leading to an increase in the prevalence of gastrointestinal nematodes and resulting in low profitability in small ruminants. Therefore, there is a need to develop alternative control strategies to reduce the prevalence and damage caused by these parasites in extensive systems. One of these strategies involves plant extracts and their secondary metabolites, which have shown antiparasitic properties. The main aim of the present study was the evaluation of *Artemisia cina (A. cina)* foliage to perform an *n*-hexane extract and cinaguaiacin as secondary metabolite (mixture of 63% of 3′-demethoxy-6-O-demethylisoguaiacin and 37% norisoguaiacin), previously identified by chromatography technique and relative expression of three antioxidant enzyme genes of infective *Haemonchus contortus* larvae (L_3_). The results showed upregulation of glutathione peroxidase (GPx) and catalase (CAT), and decreased expression of superoxide dismutase (SOD) genes after exposure to *H. contortus* L_3_ to *n*-hexane extract of *A. cina*. Furthermore, cinaguacin displayed up- and downregulation of GPx and superoxide dismutase genes, respectively. These data suggest the active function of reactive oxidative species (ROS) genes of *H. contortus* L_3_ exposed by the extract of *A. cina* and cinaguaiacin to induce the larve death. In this sense, both alternatives could be promising to mitigate resistance to anthelmintic drugs.

## Simple Summary

Anthelmintic resistance is a significant problem affecting animal production parameters. In this regard, there is a need to develop alternative control strategies to reduce the prevalence and damage caused by these parasites in extensive systems. One such strategy is plant extracts and their secondary metabolites, as they have demonstrated antiparasitic action. Therefore, the present study aimed to evaluate the relative expression of glutathione peroxidase (GPx), catalase (CAT), and superoxide dismutase (SOD) genes of third instar larvae of *H. contortus* exposed to *n*-hexane extract of *A. cina* and cinaguaiacin. The results showed upregulation of GPx and CAT, and decreased expression of SOD genes after exposure of *H. contortus* L_3_ with *n*-hexane extract of *A. cina*. Furthermore, cinaguacine showed up- and downregulation of GPx and SOD genes, respectively. These data suggest the active function of *H. contortus* L_3_ ROS genes, exposed by *A. cina* and cinaguayacin extract, to induce larval death. In this sense, both alternatives could be promising for mitigating resistance to anthelmintics.

## 1. Introduction

*Haemonchus contortus*, commonly known as the “barber’s pole worm”, is a trichostrongylid parasite and one of the most significant internal parasites affecting small ruminants, leading to substantial economic losses. The parasite’s infective stage occurs when animals ingest contaminated feed containing numerous third-stage larvae. These larvae enter the abomasum and may migrate to the gastric glands, where they can remain dormant. After developing into adults, the parasites return to the abomasal surface and attach via a buccal tooth. Their eggs are excreted in the feces, completing the life cycle. *H. contortus* is a blood-feeding parasite that causes severe anemia, hypoproteinemia, and subsequent edema. The intermandibular oedema is due to the vascular leakage caused by hypoproteinemia [1,2]. Due to factors such as large effective population sizes, the excessive use of anthelmintics, and high levels of genetic diversity, *H. contortus* has rapidly developed resistance to a wide range of antiparasitic drugs [3].

*Artemisia cina (A. cina)*, a plant known for its traditional medicinal properties, has shown promising anthelmintic effects against gastrointestinal parasites in sheep, such as *Haemonchus contortus (H. contortus)*. The active compounds in *A. cina*, including santonin, have been studied for their ability to disrupt the lifecycle of these parasites. When administered to infected sheep, extracts from *A. cina* inhibit the development and reproduction of *H. contortus*, thereby reducing the parasitic burden [4]. In this sense, this plant provides a natural alternative to synthetic anthelmintics, which are becoming increasingly ineffective due to the development of parasite resistance. It offers a sustainable and viable approach to managing parasitic infections in livestock [5,6,7].

The mechanism by which *A. cina* has shown anthelmintic effects involves interference with the parasite’s neuromuscular system, leading to paralysis and eventual expulsion by the host [8]. Studies conducted in our laboratory have demonstrated that treatments using *A. cina* can significantly lower egg counts in sheep feces, indicating a reduction in parasite load [9]. This sets an attractive precedent for the use of *A. cina* as an anthelmintic in organic and sustainable farming practices, as it could result in fewer risks of chemical residues in meat and wool [10].

The Asteraceae family, (to which the genus *A. cina* belongs), which includes a variety of medicinal plants, exhibits antiparasitic activity through several mechanisms of action [11]. One of the key ways these plants combat parasites is by producing bioactive compounds such as sesquiterpene lactones, flavonoids, and polyacetylenes [12]. These compounds interfere with the metabolic processes of parasites, leading to impaired growth, development, and reproduction. Sesquiterpene lactones, for instance, can disrupt the integrity of the cell membranes of parasites or inhibit essential enzymes, causing cellular damage and ultimately leading to death. Meanwhile, flavonoids and polyacetylenes contribute to antiparasitic activity by generating oxidative stress within the parasites, which leads to apoptosis, or programmed cell death. These multifaceted actions make Asteraceae plants an effective natural option for controlling a wide range of parasitic infections in both humans and animals [13,14]. 

Enzymes like glutathione peroxidase (GPx), catalase (CAT), and superoxide dismutase (SOD) play crucial roles in parasites’ metabolism and detoxification processes. These enzymes are part of the antioxidant defense system that helps protect parasites from oxidative stress caused by reactive oxygen species (ROS) generated during metabolic activities [15]. Specifically, GPx reduces hydrogen peroxide and lipid peroxides to water and alcohol, using glutathione as a substrate, thus preventing cellular damage. As for CAT, it decomposes hydrogen peroxide into water and oxygen, efficiently reducing oxidative stress. Lastly, SOD converts superoxide radicals into hydrogen peroxide and molecular oxygen, providing additional protection against oxidative damage. These enzymes work in synergy to maintain the redox balance within the parasite, ensuring its survival and ability to thrive in the host environment [16,17].

Lignans, a group of polyphenolic compounds found in various plants, have been shown to influence the activity of antioxidant enzymes, including glutathione peroxidase, catalase, and superoxide dismutase. The possible mechanism of action of lignans involves their ability to enhance the expression and activity of these enzymes, thereby bolstering the antioxidant defense system [18,19]. Furthermore, lignans can scavenge free radicals and reduce oxidative stress, indirectly supporting the function of glutathione peroxidase by maintaining adequate levels of reduced glutathione [19]. By modulating the activity of these enzymes, lignans contribute to cellular protection against oxidative damage and promote overall metabolic stability, which can be particularly beneficial in mitigating oxidative stress-related conditions in both host and parasite systems [16,20]. This study aimed to evaluate the relative expression of glutathione peroxidase, catalase, and superoxide dismutase genes of third-stage larvae of *H. contortus* exposed to *n*-hexane extract of *A. cina* and cinaguaiacin, a secondary metabolite of *A. cina* consisting of a mixture of 3′-demethoxy-6-O-demethylisoguaiacin and norisoguaiacin.

## 2. Materials and Methods

### 2.1. Plant Material

Aerial parts of *A. cina* were obtained from a commercial laboratory (Millennium lab^®^, Mexico City), where they were harvested in the pre-flowering stage under standardized conditions, such as temperature (24 °C), pH of the soil (6.3), and humidity (80%). Finally, the taxonomic identification was performed by Dr. Alejandro Torres-Montúfar with voucher No. 11967. The plant material was dried at room temperature (24–26 °C) in a press for 15 days.

### 2.2. n-Hexane Extract and Lignans

One kilogram of vegetal material was dried at room temperature and macerated with *n*-hexane for 48 h. The *n*-hexane extracts were filtered using a Whatman No. 4 paper (Millipore, Dublin, Ireland), and the solvent was removed by low-pressure distillation using a rotary evaporator (Heidolph Laborota 4000, Heidolph Instruments, Schwabach, Germany) until complete dryness.

The presence of lignans was confirmed by high-performance liquid chromatography (HPLC). Briefly, the *n*-hexane extract was analyzed by normal-phase chromatography using a mobile phase of *n*-hexane; ethyl acetate (70:30), and the active compounds 3′-demethoxy-6-O-demethylisoguaiacin and norisoguaiacin (cinaguaiacin) were identified and isolated. The molecular weight was confirmed by mass spectrometry.

### 2.3. Mass Spectrometry Lignans Evaluation

The separated lignans were analyzed by triple/quadruple mass spectrometry (TQ/MS; ACQUITY Ultraperformance coupled to an MS Xevo electrospray source, Waters Corp., Milford, MA, USA). The system utilized positive electrospray ionization mode (ESI+) with *n*-hexane HPLC grade as the solvent. The spectra were obtained over the range of 20–400 V.

### 2.4. Haemonchus contortus Strain

Third-stage larvae (L_3_) of *H. contortus* were obtained from donor sheep that had been previously infected with 5000 L_3_ of the FESC UNAM strain (Cuautitlán Izcali, Mexico). Larval cultures were performed using a modified Corticelli and Lai parasitological technique [21]. Fecal samples were kept in petri dishes at 25 °C in a laboratory oven (Laboratory incubator, UNC-L-CU60, Waltham, Massachusetts, EEUU). After 15 days, the cultures were harvested, and the larvae were gathered and stored at 4 °C until use.

### 2.5. Haemonchus contortus L_3_ Relative Expression Assay

The relative expression of GPx, SOD, and catalase (CAT) enzyme genes as a biomarker of oxidative stress post-treatment with *n*-hexane *A. cina* extract and cinaguaiacin was evaluated. Total RNA extraction was performed, followed by reverse transcription quantitative PCR (RT-qPCR) methodology. The ΔΔCt analysis was performed using Qiagen’s RT2 Profiler PCR Data Analysis tool, specifically the GeneGlobe Data Analysis Center. Treatments consisted of the following: (1) *A. cina* 4 mg/mL and (2) cinaguaiacin 2 mg/mL; (3) 0.078% hydrogen peroxide as positive control and (4) Tween 20 as a negative control.

### 2.6. Total RNA Extraction

Unsheathed third-stage larvae of *H. contortus* (20,000 per well) were exposed to *A. cina n*-hexane extract and cinaguaiacin as treatments, respectively. Treatments were exposed for 24 h at a laboratory temperature of 25 °C. The treatments were performed in triplicate. After this time, the larvae were centrifuged, washed, and transferred to 1.5 mL microcentrifuge tubes containing 500 µL of TRIzol, and then kept at 4 °C for 24 h. The microcentrifuge tubes were placed in liquid nitrogen for two minutes, and afterward, larvae were ground with a pestle. This procedure was repeated six times, followed by the addition of 100 μL of chloroform (Axyspin R, Axygen Scientific, *Loughborough*, *Inglaterra*, *Reino Unido*) and centrifugation at 11,300× *g* for 15 min at 4 °C. The aqueous phase was transferred into a new tube, and 500 μL of cold isopropanol (−18 °C) was added. The mixture was then incubated for 10 min in liquid nitrogen and for 1 h at −18 °C. Then, it was centrifuged at 13,000× *g* for 15 min at 4 °C. The supernatant was removed, and 500 μL of 75% ethanol was added. Finally, the sample was centrifuged at 7500× *g* for 5 min; the supernatant was removed and allowed to dry, then resuspended in nuclease-free water (Cleaver Scientific, Rugbu, Warwickshire, UK). Total RNA (tRNA) was quantified through spectrophotometry (nanodrop, Thermo Fisher Scientific, Wilmington, DE, USA).

### 2.7. Reverse Transcriptase

To synthesize cDNA from 500 ng of total RNA (tRNA), reverse transcription (RT) was performed using a reverse transcriptase enzyme. For this, two mixtures were prepared. Mixture 1, composed of Oligo (Thermo Scientific, Waltham, MA, USA) 1 μL, dNTP (Thermo Scientific) 1 μL, nuclease-free water (Cleaver Scientific) 12 μL, and 2 μL of tRNA, was incubated in a thermal cycler (Apollo Instrumentation ATC401, Irvine, CA, USA) at 65 °C for 5 min. Then, it was kept in a refrigerated rack and mixture 2, composed of 5x buffer (Invitrogen, Nauclapan, México) 4 μL and DTT (Invitrogen) 2 μL, was added and incubated at 37 °C for 2 min. Afterward, it was placed on a refrigerated rack, and 1 μL of reverse transcriptase (Invitrogen, Waltham, MA, USA) was added. The mixture was then incubated for 50 min at 37 °C. Finally, the enzyme was inactivated by incubating at 70 °C for 15 min. The cDNA was purified using a commercial cleanup kit (e.g., Qiagen MinElute PCR Purification Kit, Beverly, MA, USA) prior to spectrophotometric quantification (Nanodrop, Irvine, CA, USA). Purity was assessed via A260/A280 ratios (acceptable range: 1.8–2.0), and only samples meeting this criterion were used for qPCR.

### 2.8. Real-Time PCR Assay

The reaction was prepared according to the protocol described in a SensiFAST^®^ commercial kit, consisting of 10 μL of SensiFASTTM SYBR No-ROX Kit, 7 μL of nuclease-free water, 1 μL (5 mM) of each oligonucleotide (T4Oligo^®^, México city, Mexico), and 1 μL of sample. The final volume reaction was 22 µL. The gene sequences were designed from sequences reported in the National Center for Biotechnology Information (https://www.ncbi.nlm.nih.gov/, (accessed on 1 January 2024)). The gene sequences were analyzed using the Primer Select Sequencer software (Primer3Plus®) and are described in Table 1.

The qPCR was performed on a Strategene Mx3005P device (Agilent Technologies, USA). The amplification conditions for qPCR were established according to what was described in the kit protocol (SensiFASTTM): an initial denaturation cycle at 95 °C for 180 s, followed by 40 cycles: denaturation at 95 °C for 10 s; the alignment temperature was specific for each pair of primers (glutathione peroxidase 58 °C; catalase 60 °C; dismutase 57 °C and β-tubulin 59 °C). After these 40 cycles, one more was added for dissociation, which included 95 °C for 10 s of denaturation and 95 °C for 10 s of extension. The Ct values of each reaction were recorded in the corresponding template for evaluation using the Qiagen GeneGlobe Data Analysis Center tool. All RT-qPCR assays were performed in triplicate.

### 2.9. Statistical Analysis

The comparative threshold (Ct) method was used for the relative expression assay. Gene expression was calculated based on the number of cycles where amplification exceeded the threshold. The data obtained were analyzed with the Qiagen^®^ GeneGlobe. The Data Analysis Center tool obtains the fold change value and *p*-value.

## 3. Results and Discussion

**Figure 1:**
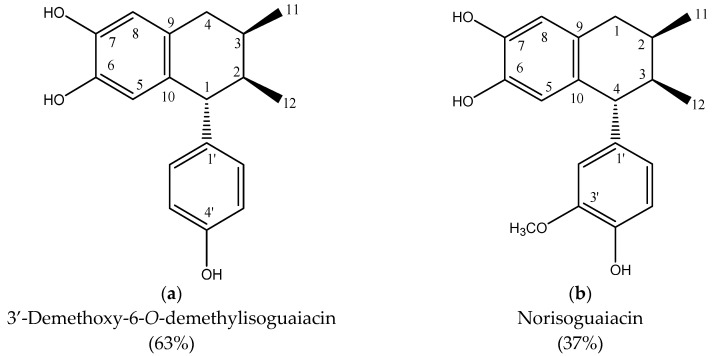
Structures of (**a**) 3′-demethoxy-6-O-demethylisoguaiacin and (**b**) norisoguaiacin isolated from *Artemisia cina*.

The structural molecules that comprise cinaguaiacin are depicted in Figure 1. Both molecules were obtained from the *n*-hexane extract of *A. cina*. These structures are chemically lignans belonging to the phytoestrogens group, and the only thing that differentiates them is that in norisoguaiacin, a methyl group is present in the C3’ position.

**Figure 2:**
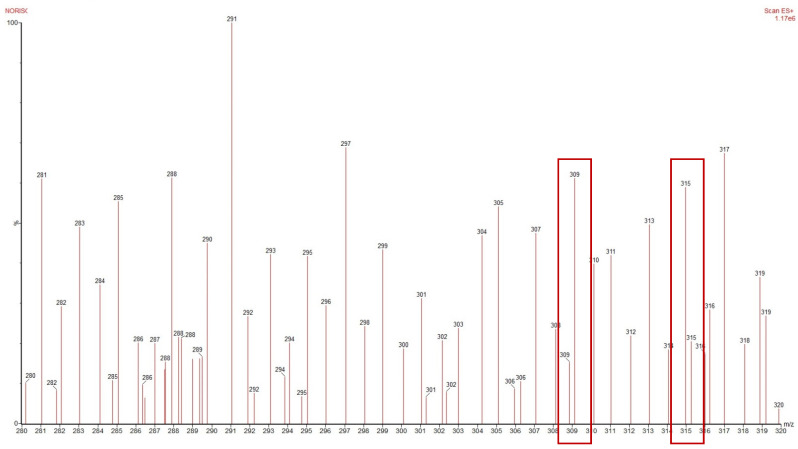
Mass spectrum of 3′-demethoxy-6-O-demethylisoguaiacin and norisoguaiacin. 3′-demethoxy-6-O-demethylisoguaiacin: [Mass + Na + 2H^+^]:309 *m*/*z* and norisoguaiacin [Mass + Na + 2H^+^]:315 *m*/*z*.

The mass spectrum, used for molecular weight confirmation of two lignans, is shown in Figure 2. In the mass spectrum, two peaks corresponding to 3′-demethoxy-6-O-demethylisoguaiacine and norisoguaiacin are observed, specifically at *m*/*z* ratios of 309 and 315, respectively.

### Haemonchus contortus L3 Relative Expression Assay

The results of the relative expression of the genes of the enzymes GPx, SOD, and CAT as biomarkers of oxidative stress are shown in Table 2. The *n*-hexane extract of *A. cina* showed significant upregulation of the GPx gene (*p* = 0.000001) and a trend towards upregulation in the CAT gene (*p* = 0.1433). Meanwhile, a trend toward downregulation of the SOD gene was observed upon treatment with *A*. *cina* extract (*p* = 0.0534). Furthermore, cinaguaiacin showed only a significant upregulation of the GPx gene (*p* = 0.0277), but there is a tendency to downregulate CAT (*p* = 0.0799) and SOD (*p* = 0.0972) gene expression. Finally, in the case of hydrogen peroxide, which was used as a control in the present study, a significant downregulation was represented in the GPx gene (*p* = 0.0208) and a tendency towards downregulation in SOD (*p* = 0.0553). In contrast, although there was an upregulation in the CAT gene, this was not significant.

Haemonchus contortus releases extracellular superoxide dismutase (Hc SODe) as a defense against reactive oxygen species (ROS) produced by the host’s immune cells [22]. This enzyme helps the parasite survive by neutralizing the oxidative stress imposed by the host’s immune response [22,23]. Lubisch et al. demonstrated that the vaccination of lambs with recombinant Hc SODe expressed in E. coli resulted in a slight reduction in worm burden, indicating a potential, albeit limited, protective effect [22,23]. Alternative treatments, such as copper edetate supplementation in *H. contortus*, activate superoxide dismutase, indicating that certain supplements can influence the activity of this enzyme [24]. Superoxide dismutase in *H. contortus* is crucial in protecting the parasite from the host’s immune response by neutralizing reactive oxygen species (ROS) [22,23,24].

The present study identified two lignans, 3-demethoxy-6-O-demethylisoguaiacin and norisoguaiacin, obtained from the *n*-hexane extract of *A. cina*. These lignans induce the relative expression of enzymes such as glutathione peroxidase and catalase in the hematophagous parasite *H. contortus*. This overexpression demonstrates that exposing the larvae to the compounds and the extract causes changes in the genes that express stress-associated enzymes, suggesting a possible mechanism of action for these compounds as anthelmintics. In this context, they stress the larvae and inhibit their ability to detoxify. Additionally, the anthelmintic activity of these molecules has been reported [9] in vitro and in vivo assays [10].

The lignans are a group of bioactive compounds found in various plants, such as those in the Asteraceae family [25]. They have been shown to influence oxidative stress by modulating the activity of key antioxidant enzymes. In the case of SOD, lignans can stimulate the expression of SOD, which plays a crucial role in converting superoxide radicals into hydrogen peroxide (H_2_O_2_) and oxygen, thereby reducing oxidative stress [26,27]. Gao et al. reported that nectandrin B, a lignan from nutmeg, has been shown to increase SOD I and II expression in aged human diploid fibroblasts, contributing to reduced intracellular reactive oxygen species (ROS) production [26]. Catalase is responsible for decomposing H_2_O_2_ into water and oxygen, preventing its accumulation and potential cellular damage [27,28]. The direct effect of lignans on catalase activity is not explicitly detailed; the overall reduction in ROS due to lignan activity suggests a supportive role in maintaining catalase function [26].

GPx uses glutathione (GSH) to reduce H_2_O_2_ and lipid peroxides, playing a vital role in protecting cells from oxidative damage [27]. Lignans, by reducing oxidative stress and ROS levels, indirectly support the activity of GPx, ensuring efficient detoxification of peroxides [26]. In the third stage of *H. contortus* larvae, the cinaguaicin and *n*-hexane extracts induce death by inhibiting detoxification and metabolism. Regarding this, Cui et al. [29] reported that the addition of *Artemisia annua* to the diet has been shown to increase the activities of CAT, GPx, and SOD in geese, enhancing their total antioxidant capacity and reducing oxidative stress markers like malondialdehyde. Also, studies on aquatic animals indicate that *A. annua* extract can boost antioxidant enzyme activities, including CAT, GPx, and SOD, thereby reducing oxidative stress [30]. The ethyl acetate fraction of *Artemisia capillaris* and *Artemisia iwayomogi* has been found to increase the activities of SOD, GPx, and CAT, contributing to their hepatoprotective and antifibrotic effects [31]. *Artemisia cina* extract has been shown to upregulate the expression of GPx in parasites like *H. contortus*, indicating its role in enhancing antioxidant defenses [32]. *Artemisia arborescens* based treatments restored the activities of SOD, CAT, and GPx in the kidneys of treated groups, reducing lipid peroxidation and providing protection against nephrotoxicity [33].

The Vanshita et al. [34] study demonstrates that quercetin induces oxidative stress in *H. contortus* by generating reactive oxygen species (ROS), particularly targeting the nervous system. This lignans with findings in the present study, where plant extracts like *Artemisia cina* also disrupted parasite redox balance by modulating antioxidant enzymes such as glutathione peroxidase (GPx) and catalase (CAT). The upregulation of these enzymes in response to quercetin suggests a compensatory mechanism by the parasite to counteract ROS-induced damage. However, the overwhelming oxidative stress ultimately leads to paralysis and death, highlighting the potential of flavonoids like quercetin as anthelmintics. This mechanism is distinct from synthetic drugs, offering a novel approach to combat resistance, as seen in the differential enzyme responses between quercetin and albendazole treatments [34].

Quercetin’s efficacy across all developmental stages of *H. contortus*—eggs, larvae, and adults—underscores its broad-spectrum potential. The larval mortality assay revealed concentration-dependent effects, with quercetin outperforming albendazole at higher concentrations. This mirrors the results of García-Coiradas et al. [35], where isolated antigens from *H. contortus* showed stage-specific immunogenicity. Quercetin’s preferential targeting of neuronal tissues, evidenced by ROS accumulation in neuropils, contrasts with albendazole’s generalized cuticular damage. Such specificity may reduce off-target effects in hosts, as mammalian neurons are structurally and functionally distinct from nematode neurons, a point further supported by the non-toxic profile of quercetin in mammals [34].

The rise of anthelmintic resistance necessitates alternative strategies, and quercetin’s natural origin and multi-target action make it a promising candidate. The present study emphasized the role of plant secondary metabolites in overcoming resistance, as seen with *A. cina* lignans. Similarly, quercetin’s ability to alter stress-response enzymes (CAT, SOD, GPx) suggests it disrupts multiple metabolic pathways, reducing the likelihood of resistance development. Future studies should explore synergistic effects with existing drugs, as Borges et al. [35] noted enhanced ivermectin efficacy when combined with quercetin. Additionally, formulation improvements could enhance quercetin’s bioavailability and potency, paving the way for field applications in livestock.

Similar results were reported by Goel et al. [19], who reported that cuminaldehyde (CA), a monoterpenoid from cumin, induces oxidative stress in *H. contortus*, leading to physical damage and death of the parasite. Both studies underscore the role of oxidative stress as a key mechanism of action for these compounds. While our study focused on damage induced by lignans from *A. cina*, Goel et al. [19] provided detailed evidence of CA’s ability to elevate reactive oxygen species (ROS) and antioxidant enzyme activities, such as catalase and superoxide dismutase, in *H. contortus*. Together, these findings suggest that plant-derived compounds, whether from *Artemisia cina* or cumin, can effectively target multiple life stages of *H. contortus* through oxidative pathways, offering promising alternatives to conventional anthelmintics.

Moreover, the comparative analysis of these studies reveals broader implications for combating anthelmintic resistance. The present study emphasizes the need for further in vivo studies to validate the lignans’ safety and efficacy in the ovine model, while Goel et al. [19] highlight CA’s preclinical potential, noting its superior performance in inducing oxidative damage over albendazole. Both studies advocate for exploring plant-based anthelmintics as sustainable solutions, particularly in regions where resistance to synthetic drugs is prevalent. Future research could investigate synergistic effects of combining these compounds or their derivatives to enhance efficacy and delay resistance development. Additionally, the methodologies employed—such as egg hatch assays and larval mortality tests—provide a robust framework for evaluating novel anthelmintics. By integrating these findings, the scientific community can advance toward developing safer, more effective treatments for gastrointestinal nematode infections in livestock, addressing both economic and health challenges in agriculture.

The study by Grosskopf et al. [18] evaluated the effects of copper edetate supplementation on *Haemonchus contortus*-infected sheep, demonstrating that while the treatment did not significantly reduce parasite oviposition or EPG counts, it enhanced superoxide dismutase (SOD) activity, an antioxidant enzyme critical for mitigating oxidative stress. This aligns with the findings of our study, where we reported that hexane extract of *Artemisia cina* inducing oxidative stress in *H. contortus*. Both studies underscore the importance of oxidative stress as a mechanism for combating parasitic infections, though they target different pathways; copper edetate indirectly modulates host antioxidant defenses, while lignans and hexane extract directly induce oxidative damage in the parasite.

However, Grosskopf et al. [18] also noted that copper edetate caused mild inhibition of cholinesterase activities (AChE and BChE), which could affect host neuromuscular and immune functions. In contrast, the present study focused on the parasite-specific toxicity of methyl chavicol without apparent host toxicity, suggesting a potential advantage for plant-derived compounds in minimizing adverse effects. Together, these studies highlight the following dual challenge of developing effective anthelmintics: optimizing parasite lethality while preserving host health. Future research could explore synergistic approaches, such as combining copper edetate’s immune-modulating properties with lignans’ direct oxidative effects, to enhance efficacy and reduce resistance risks. Additionally, comparative in vivo studies are needed to evaluate these interventions’ long-term safety and practical applicability in livestock production systems.

## 4. Conclusions

The *n*-hexane extract of *A. cina* showed significant upregulation of the GPx gene and a trend towards upregulation in the CAT gene. Meanwhile, a trend toward downregulation of the SOD gene was also observed upon treatment with *A. cina* extract. Contrarily, cinaguacin showed only a significant upregulation of the GPx gene, but there was a tendency to downregulate CAT and SOD gene expression. Therefore, these modifications in the expression of antioxidant enzymes in *H. contortus* larvae are directly related to the mechanism of action of lignans, which causes the larvae’s death. This proposed mechanism of anthelmintic action may help to understand the functioning of the *n*-hexane extract of *A. cina* and cinaguacin and propose new viable treatments to mitigate the problems of resistance to anthelmintics.

## Figures and Tables

**Table 1:** Design of gene sequence for RT-qPCR.

Gene	Genebank Access Number	Tm	Sequence	Amplicon Size (bp)
Glutathione peroxidase	AY603337	58 °C	Fw: 5′-TGACGTCAACGGAGAGAACC-3’	189
			Rv: 5′-GTTGGATGGAAACGGAAGCG-3’	
Catalase	AY603335.1	60 °C	Fw: 5′-CACCGTGTTTCGTTCGCTTT-3’	184
			Rv: 5′-TGGGTGTGGATGAAGTTCGG-3’	
Superoxide Dismutase	MT015608.1	57 °C	Fw: 5′-GCAGCGTATCCGGTCTACAA-3’	121
			Rv: 5′-CCCGCTCCAAGACAACCATT-3	
β-tubulin	FJ981644.1	59 °C	Fw: 5′-GGGCAAAAGGTCACTACACA-3’	229
			Rv: 5′-TCGGAAACCTTTGGTGAAGG-3’	

**Table 2:** Relative expression of glutathione peroxidase, catalase, and superoxide dismutase genes on third larvae stage of *Haemonchus contortus* after 24 h exposed to *n*-hexane extract and 3′-demethoxy-6-O-demethylisoguaiacin and norisoguaiacin (cinaguaiacin) obtained from *Artemisia cina*.

	Hydrogen Peroxide		Β-Tubuline		*n*-Hexane Extract		Cinaguaiacin		Tween 20	
Gene	Fold Change	*p* Value	Fold Change	*p* Value	Fold Change	*p* Value	Fold Change	*p* Value	Fold Change	*p* Value
Glutathione peroxidase (GPx)	0.00 *	**0.0208**	1	0	113.64 *	**<0.0001**	0.05 *	**0.0277**	2.38	0.08
Catalase (CAT)	1.05	0.4688	1	0	**9.6**	0.1433	*0.27*	0.0799	0	0
Superoxide Dismutase (SOD)	*0.05*	0.0553	1	0	*0.27*	0.0534	*0.33*	0.0972	*0*	*0*

**Bold** = Upregulation of the expression level; *Italics* = Downregulation of the expression level; * Significant difference (*p* < 0.05).

## Data Availability

The databases used and analyzed during the current study are available from the corresponding author.

## Abbreviations

The following abbreviations are used in this manuscript:

*A. cina*	*Artemisia cina*
*H. contortus*	*Haemonchus contortus*
GPx	Glutathione peroxidase
CAT	Catalase
SOD	Dismutase superoxide
MDA	Malondialdehyde
